# Adenovirus E1A Activation Domain Regulates H3 Acetylation Affecting Varied Steps in Transcription at Different Viral Promoters

**DOI:** 10.1128/JVI.00805-18

**Published:** 2018-08-29

**Authors:** Emily Hsu, Mario A. Pennella, Nathan R. Zemke, Carol Eng, Arnold J. Berk

**Affiliations:** a Molecular Biology Institute, University of California Los Angeles, Los Angeles, California, USA; b Department of Microbiology, Immunology, and Molecular Genetics, University of California, Los Angeles, California, USA; International Centre for Genetic Engineering and Biotechnology

**Keywords:** CBP, CREBBP, EP300, adenovirus E1A, adenovirus transcription, histone acetylation, p300

## Abstract

How histone acetylation promotes transcription is not clearly understood. Here, we confirm an interaction between p300 and the adenovirus 2 large E1A activation domain (AD) and map the interacting regions in E1A by observing colocalization at an integrated *lacO* array of fusions of LacI-mCherry to E1A fragments with YFP-p300. Viruses with mutations in E1A subdomains were constructed and analyzed for kinetics of early viral RNA expression and association of acetylated H3K9, K18, K27, TBP, and RNA polymerase II (Pol II) across the viral genome. The results indicate that this E1A interaction with p300 is required for H3K18 and H3K27 acetylation at the E2early, E3, and E4 promoters and is required for TBP and Pol II association with the E2early promoter. In contrast, H3K18/27 acetylation was not required for TBP and Pol II association with the E3 and E4 promoters but was required for E4 transcription at a step subsequent to Pol II preinitiation complex assembly.

**IMPORTANCE** Despite a wealth of data associating promoter and enhancer region histone N-terminal tail lysine acetylation with transcriptional activity, there are relatively few examples of studies that establish causation between these histone posttranslational modifications and transcription. While hypoacetylation of histone H3 lysines 18 and 27 is associated with repression, the step(s) in the overall process of transcription that is blocked at a hypoacetylated promoter is not clearly established in most instances. Studies presented here confirm that the adenovirus 2 large E1A protein activation domain interacts with p300, as reported previously (P. Pelka, J. N. G. Ablack, J. Torchia, A. S. Turnell, R. J. A. Grand, J. S. Mymryk, Nucleic Acids Res **37:**1095–1106, 2009, https://doi.org/10.1093/nar/gkn1057), and that the resulting acetylation of H3K18/27 affects varied steps in transcription at different viral promoters.

## Abstract (precis)

Despite a wealth of data associating promoter and enhancer region histone N-terminal tail lysine acetylation with transcriptional activity, there are relatively few examples of studies that establish causation between these histone posttranslational modifications and transcription. While hypoacetylation of histone H3 lysines 18 and 27 is associated with repression, the step(s) in the overall process of transcription that is blocked at a hypoacetylated promoter is not clearly established in most instances. Studies presented here confirm that the adenovirus 2 large E1A protein activation domain interacts with p300, as reported previously (P. Pelka, J. N. G. Ablack, J. Torchia, A. S. Turnell, R. J. A. Grand, J. S. Mymryk, Nucleic Acids Res **37:**1095–1106, 2009, https://doi.org/10.1093/nar/gkn1057), and that the resulting acetylation of H3K18/27 affects varied steps in transcription at different viral promoters.

## INTRODUCTION

**FIG 1:**
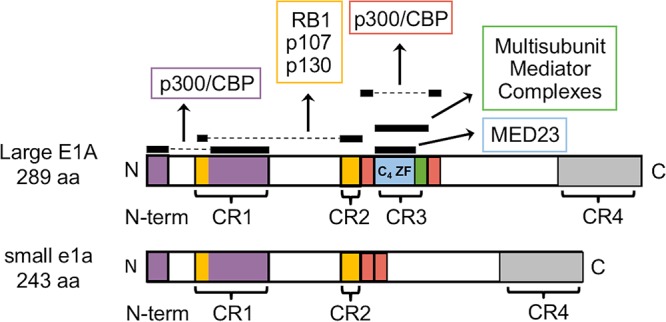
Small and large E1A protein interactions. E1A interaction maps for large E1A (289 aa) and small e1a (243 aa). Colored portions and horizontal black bars above them represent regions of E1A that interact with the cellular protein indicated by the associated arrowhead pointing to a box containing the name of the cellular protein. Dashed lines connect conserved regions of E1A that bind the indicated targets together. Conserved regions (CR) 1 to 4 are indicated in brackets. Results presented below indicate that the red regions interact with p300 and that the green region at the C-terminal end of CR3 is required for binding multisubunit mediator complexes.

Proteins encoded in early region 1A (E1A) of human adenoviruses (Ad) have been studied extensively as model transcriptional regulators to uncover molecular mechanisms that control viral and cellular gene expression. Upon infection, E1A is the first viral region transcribed, and E1A pre-mRNA is alternatively spliced into mRNAs, designated 13S, 12S, and 9S mRNAs, as well as the less abundant 10S and 11S mRNAs (1). The 13S and 12S transcripts are generated from two 5′ splice sites, separated by 138 bp and a common 3′ splice site, and are most abundant early after infection and in transformed cells. They encode the 289-amino-acid (aa) large E1A and the 243-aa small e1a proteins, respectively (2). E1A proteins do not bind directly to DNA and exert their considerable effects on gene expression by interacting with cellular proteins through sequences conserved throughout primate adenovirus species and serotypes, designated conserved regions 1 to 4 (CR1-CR4) (3, 4) (Fig. 1).

Large and small E1A both contain the N-terminal CR1 and CR2, which interact with the closely related nuclear lysine acetyltransferases CBP/p300 and RB family proteins (RB1, p107, and p130) to reprogram host cell gene expression and induce S phase in quiescent host cells (5, 6) (Fig. 1). The small e1a N-terminal ∼12 aa and aa 54 to 82 of CR1 bind the TAZ2 domain of CBP/p300 (7). p300/CBP is primarily responsible for mediating the acetylation of histone H3 at lysines 18 (H3K18) and 27 (H3K27), which are generally associated with promoters of actively transcribed regions (8, 9). The association of p300/CBP with small e1a inhibits H3K18/27 acetylation to about 30% the level in uninfected cells (5). This is thought to be the primary mechanism by which adenovirus represses host cell genes that would otherwise interfere with productive viral infection (5, 8).

While small e1a has been well characterized in the context described above, large E1A is known to modulate viral infection additionally by activating transcription from early adenovirus promoters E1A and E1B and, to a greater extent, E2early, E3, and E4 (10–13). Large E1A is primarily responsible for transcriptional activation from these promoters. Although small e1a is a much weaker activator of these promoters than large E1A, small e1a does activate transcription significantly compared to that of the E1A deletion mutant *dl*312 (13). The E1A activation domain includes CR3 [E1A(aa 140–185)], a region that is unique to large E1A. When fused to the GAL4 DNA-binding domain (DBD), E1A(aa 121–223), including CR3, functions as a strong activation domain (14). Further studies using Gal4-DBD fusions to progressive N- and C-terminal deletions of E1A(aa 121–223) suggested that the minimal region required for activation is aa 141 to 178, closely overlapping CR3 (15). Six tandem glutamic acid-proline repeats (EP_6_) just C terminal to CR3 (aa 189 to 200; designated AR1) were later shown to be essential for activation of early viral promoters by native E1A (not fused to the Gal4-DBD) in transient-transfection assays (16, 17).

In an early model of the mechanism of E1A activation of early viral promoters, the E1A activation domain was functionally divided into two distinct subdomains: a C_4_ Zn-finger region that makes contact with the basal transcriptional machinery and a C-terminal region (aa 183 to 188) implicated in promoter binding via association with host cell sequence-specific transcription factors (14, 15, 18, 19). Importantly, the large E1A activation domain, E1A(aa 121–123), binds human multisubunit mediator complexes, key coactivators required for regulating transcription by RNA polymerase II (Pol II) (20, 21). Affinity columns of E1A(aa 121–223) and amino acid substitution mutants that activate transcription were found to bind directly to the human homolog of Caenorhabditis elegans Sur2, now designated MED23, a mediator subunit located in the tail domain of the human mediator complex (22). Mutant E1As with amino acid substitutions in the C_4_ Zn-finger region (Fig. 1) that are defective for activation (18) did not bind MED23, demonstrating that MED23 associates with the Zn-finger subdomain of CR3 (20). Further work confirmed this CR3-MED23 interaction *in vivo* (23) and demonstrated its ability to stimulate Pol II preinitiation complex (PIC) assembly on promoter DNA *in vitro* (24).

While it was initially thought that activation by E1A was primarily due to its association with the mediator complex, Pelka et al. proposed that an additional E1A interaction with p300/CBP also contributes to E1A activation (25). This group reported that transcriptional activation by E1A(aa 139–204) was inhibited by overexpression of small e1a, dependent on the interaction of small e1a with p300/CBP. This result was interpreted to indicate that small e1a antagonizes transcriptional activation by large E1A through its well-characterized binding to the CBP/p300 TAZ2 domain via the E1A N terminus and CR1 (7). Further, p300/CBP also was observed to bind E1A(aa 139–204), although it did so more weakly than the well-characterized sites of E1A interaction at the N terminus and CR1 (25).

To corroborate this conclusion by an independent method, we analyzed p300 interaction with E1A(aa 121–223) *in vivo* by confocal microscopy of green fluorescent protein (GFP)-derivative tagged proteins. We constructed Ad mutants with multialanine substitutions in E1A regions observed to be required for the interaction with p300 in this *in vivo* protein-protein interaction assay and analyzed transcription from early viral promoters after infection of human primary airway epithelial cells. Additionally, we used these same methods to further characterize the association of multisubunit mediator complexes with the E1A activation domain and propose a new explanation for the requirement of the CR3 C-terminal invariant region (aa 183 to 188) for activation. Using chromatin immunoprecipitation sequencing (ChIP-seq), we analyzed the association of modified histones, TBP, and Pol II with the early viral promoters after infection with wild-type (wt) and Ad5 mutants. We found that eliminating the interaction between the large E1A activation domain and p300 prevented H3K18/27ac at the adenovirus E2early, E3, and E4 promoters during the early phase of infection. The consequences of this failure to acetylate H3K18/27 in these promoter regions for PIC assembly and transcription were different at each of these viral promoters.

## RESULTS

### Acidic regions flanking E1A CR3 are required for p300 binding to the E1A activation domain, and amino acids 179 to 189 are required for E1A binding to multisubunit mediator complexes *in vivo*.

**FIG 2:**
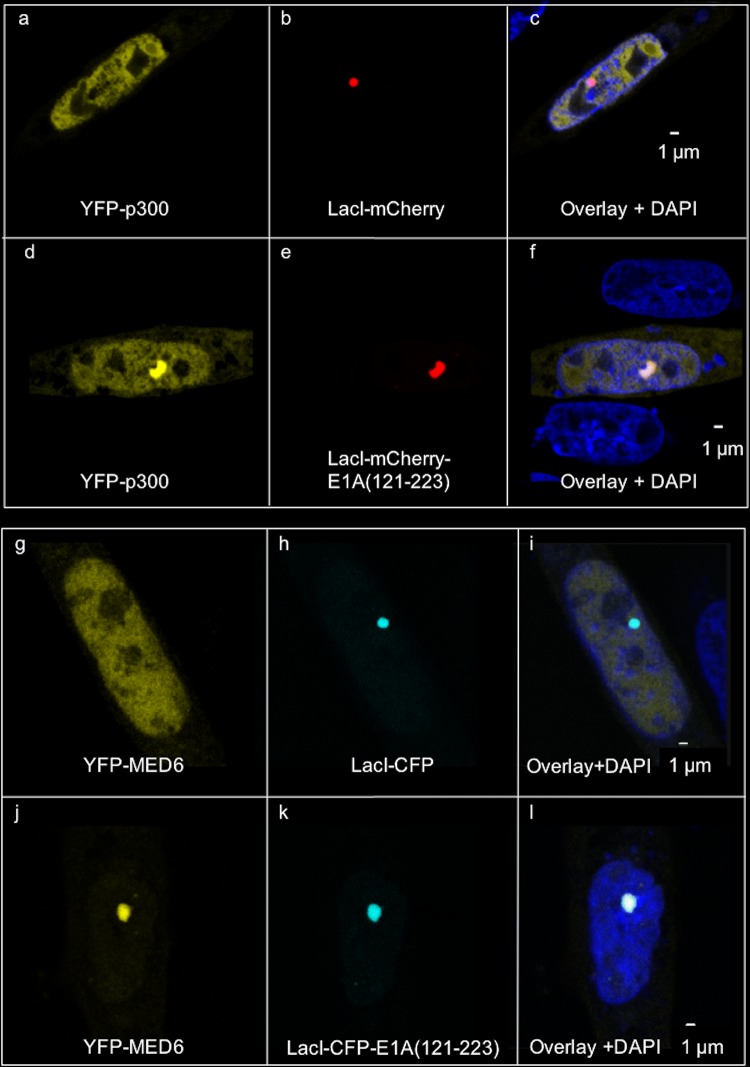
*In vivo* colocalization assays for association of fragments of large E1A with p300 and MED6. (Upper) Confocal micrographs of YFP and mCherry fluorescence in CHO-A03.1 cells cotransfected with expression vectors for YFP-p300 and LacI-mCherry (a to c) or YFP-p300 and LacI-mCherry-E1A(121–223) (d to f). (Lower) As described for upper images but with expression vectors for YFP-MED6 and LacI-CFP (g to i) or YFP-MED6 and LacI-CFP-E1A(121–223) (j to l).

Pelka et al. reported that p300 interacts with large E1A(aa 139–204), a region lacking the well-characterized large and small E1A p300/CBP binding site formed from the E1A N-terminal ∼12 aa plus E1A(aa 54–82) in CR1 (7, 25). To confirm and extend this conclusion, we analyzed protein-protein interactions *in vivo* by confocal fluorescence microscopy of proteins fused to differently colored GFP derivatives. Expression vectors for YFP-p300 and LacI-mCherry alone or LacI-mCherry fused to an E1A fragment consisting of aa 121 to 223, E1A(aa 121–223), were transfected into CHO-A03.1 cells. These cells contain an amplified region of DNA with ∼10,000 *lac* operator sites (*lacO*) integrated into a single region of one chromosome (26). Expression of mCherry-tagged Lac repressor (LacI-mCherry) resulted in clear association of this fusion protein with the *lacO* array, as observed by a localized nuclear mCherry signal (Fig. 2b and c). Coexpressed YFP-p300 did not colocalize with LacI-mCherry at the *lacO* array (Fig. 2a to c). However, when coexpressed with LacI-mCherry-E1A(121–223), YFP-p300 did colocalize with the *lacO* array (Fig. 2d to f), confirming the interaction of E1A(aa 121–223) with p300 *in vivo* (25).

**FIG 3:**
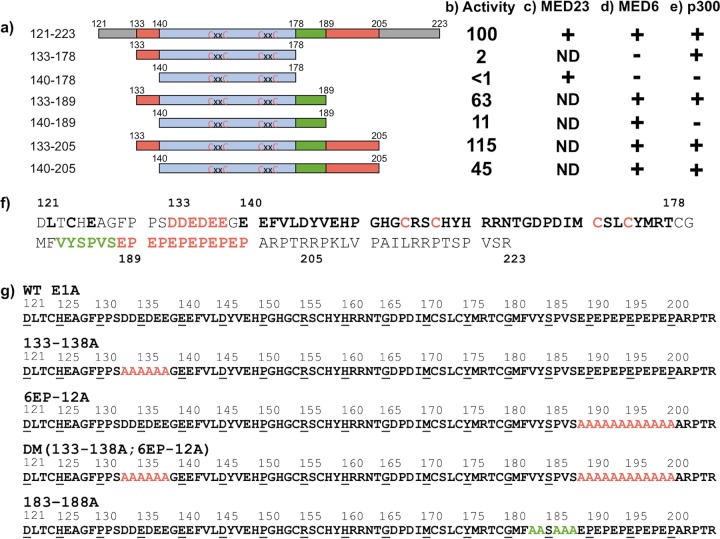
Summary of E1A deletion constructs and Ad5 mutant sequences. (a) Diagram of E1A regions fused to LacI used for colocalization and transient-transfection activation assays. E1A(aa 133–139) and E1A(aa 190–205) containing acidic peptides are shown in red. E1A(aa 179–189) is shown in green. The C_4_ Zn-finger region (aa 140 to 178) is shown in light blue, with cysteines shown in red. (b) Activation by LacI-mCherry fusions to E1A fragments in a transient-transfection luciferase reporter assay in CHO cells. The luciferase reporter had eight *lacO* sites upstream of the E1B promoter. Values are relative (rel.) to activity of LacI-mCherry-E1A(121–223). (c) Colocalization of LacI-CFP-E1A fragments with YFP-MED23. ND (not done) indicates E1A fragments that were not assayed for colocalization. (d) Colocalization of LacI-CFP-E1A fragments with YFP-MED6. (e) Colocalization of LacI-mCherry-E1A fragments with YFP-p300. (f) The amino acid sequence of wt Ad5 E1A(121–223). The LxCxE motif of CR2 (aa 122 to 126) is in boldface. Acidic peptide sequences (aa 133 to 138, DDEDEE, and aa 189 to 200, EPEPEPEPEPEP) are shown in red. The invariant sequence in the interval E1A(aa 183–188) is in green. The C_4_ Zn-finger region (aa 140 to 178) is in boldface, with cysteines in red. (g) Sequences of Ad5 mutants from E1A(aa 121–223), with alanine substitutions in red or green.

To identify the amino acids of large E1A required for this specific association with p300, we assayed colocalization of YFP-p300 with LacI-mCherry fused to deletions of E1A(aa 121–223) (Fig. 3a). Fusion proteins that retained either one of two highly acidic peptides flanking CR3, aa 133 to 138 (DDEDEE) or aa 189 to 200 (EPEPEPEPEPEP) (red in Fig. 3a and f), continued to colocalize with YFP-p300, but E1A(aa 140–189) and E1A(aa 140–178), which have both acidic peptides deleted, did not (Fig. 3e and Table 1). These results suggest that both acidic regions flanking CR3 contribute to p300 binding and that at least one of these acidic regions is required for p300 binding to the E1A activation domain, as detected in this *in vivo* colocalization assay.

**TABLE 1:** Colocalization score of LacI-mCherry or LacI-mCherry-E1A fragment with YFP-MED6, YFP-MED23, and YFP-p300

Labeled protein	Colocalization score (%; no. of cells scored) for indicated E1A fragment fused to tagged LacI^*a*^:							
	LacI	121–223	133–178	133–189	133–205	140–178	140–189	140–205
YFP-MED6	5; 22	79; 24	30; 27	80; 20	70; 20	26; 35	84; 25	76; 25
YFP-MED23	23; 30	100; 14	ND	ND	ND	100; 15	ND	ND
YFP-p300	0; 21	86; 21	64; 21	86; 15	73; 20	3; 23	4; 22	64; 20

a ND, not done.

We further characterized the known association of E1A and mediator complexes. *In vivo* colocalization, assayed as described above, was again used to observe the association of E1A deletion constructs with mediator subunit MED23, located in the tail of the mediator (22) and bound directly by large E1A (20), and MED6, located in the head domain of the mediator (22) (Fig. 2g to l). We interpreted colocalization of an E1A fragment with YFP-MED6 to indicate that multisubunit mediator complexes associate with the fragment. Consistent with previous results, E1A(aa 121–223) associated with MED23 (20, 27). However, in contrast to E1A(aa 121–223), the E1A C_4_ Zn-finger region alone (aa 140 to 178) (28) associated with MED23 but was greatly reduced in its association with MED6, as was E1A(aa 133–178) (Fig. 3c and d and Table 1). Interestingly, binding of both MED23 and MED6 required aa 140 to 189 (Fig. 3c and d and Table 1). These results indicate that E1A(aa 140–178) interacts with MED23, while the additional residues 179 to 189 (green in Fig. 1 and 3a) are required to bind multisubunit mediator complexes containing the assayed MED6 head subunit.

### Both p300 and multisubunit mediator binding by E1A are necessary for high levels of activation in a transient-transfection assay.

Several groups previously have defined the boundaries of the CR3 activation domain using transient-transfection reporter assays. E1A(aa 140–178), including a C_4_-type zinc finger, is essential for activation (14, 15, 18). In addition, peptide regions that closely overlap those that we propose to contribute to p300 and multisubunit mediator binding (aa 190 to 205 and aa 179 to 189, respectively) are required for high levels of activation (16–18). To determine if activation by the LacI-mCherry-truncated E1A fragments analyzed here is consistent with previously demonstrated activation by E1A fragments in transient-transfection assays, we assayed activation by these LacI fusions using a luciferase reporter with eight *lacO* sites upstream of the E1B promoter (Fig. 3b) (29). These assays were done in CHO-K1 cells, the parental cells for the A03.1 cells used in the colocalization assays.

Although deletion of either the N- or C-terminal acidic region of E1A(aa 133–205) did not affect p300 binding in our *in vivo* colocalization assay, E1A(aa 133–189) and E1A(aa 140–205), each lacking one of the two acidic peptides flanking CR3, displayed partial defects in activation, with ∼50% of the activity of E1A(aa 121–223) (Fig. 3b). Deletion of both acidic regions and the consequential complete loss of p300 binding by this region of E1A(aa 140–189) (Fig. 3e and Table 1) resulted in a much greater defect in activation, with only ∼10% the activity of E1A(aa 121–223) (Fig. 3b). The greatest defect in activation resulted from loss of association with multisubunit mediator complexes. E1A(aa 133–178) and E1A(aa 140–178), both lacking aa 179 to 189, had <3% the activity of E1A(aa 121–223), regardless of their ability to associate with p300 (Fig. 3b to e). These results indicate that E1A binding to a multisubunit mediator complex is essential for activation by E1A(aa 121–223), and that the additional interaction with p300 further stimulates transcription ∼10-fold in this transient-transfection assay.

### During adenovirus infection, transcription from each of the viral early promoters responds differently to multialanine substitutions in subdomains of the E1A activation domain.

**FIG 4:**
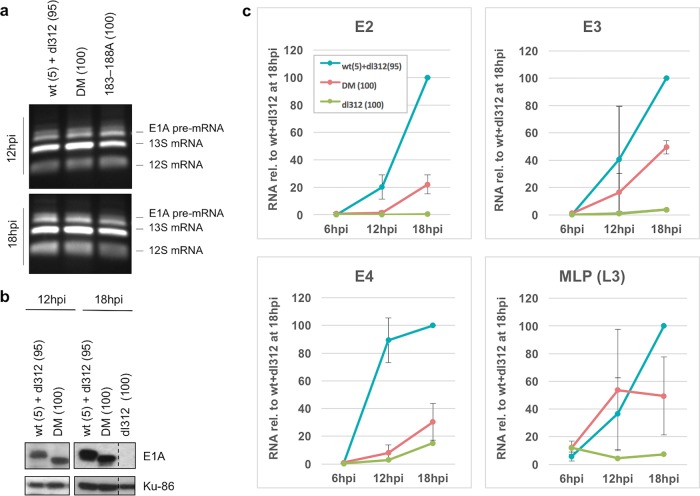
Activation of early viral promoters and MLP by wt and DM E1A. (a) RT-PCR using primers spanning the major E1A splice sites with total cell RNA from HBTECs coinfected with the wt E1A-expressing virus at an MOI of 5 and *dl*312 at an MOI of 95 or infected with the DM or 183-188A vectors alone at an MOI of 100. The lengths of the pre-mRNA, 12S, and 13S transcripts are indicated. Only the region of the gel with visible bands is shown. (b) Western blot showing wt and DM large E1A protein levels at 12 and 18 h p.i. HBTEC cells were coinfected with the wt E1A-expressing virus at an MOI of 5 and *dl*312 at an MOI of 95 or infected with the DM virus alone at an MOI of 100. Ku-86 protein levels were used as loading controls. (c) Relative E2, E3, E4, and L3 RNA in infected HBTECs at the indicated times p.i., as assayed by qRT-PCR. HBTECs were infected with the *dl*312, wt and *dl*312, and DM viruses at the same MOIs as those described for panel a. Values are plotted relative to those of wt and *dl*312 at 18 h p.i. Data are represented as averages ± standard deviations (SD) from three independent experiments.

To determine whether the decreased activation by LacI-E1A deletions in transient-transfection assays is recapitulated by comparable E1A mutants during adenovirus infection, we constructed Ad5 mutants with multialanine substitutions in E1A regions necessary for maximal activation in the transient-transfection assay (Fig. 3g). These included the 133–138A and 6EP–12A mutants, with alanine substitutions in the N- and C-terminal acidic regions (Fig. 3a and g, red regions), respectively, the double mutant (DM), with alanine substitutions in both acidic regions, and the 183 to 188A mutant, with alanine substitutions in the invariant region required for binding multisubunit mediator complexes (Fig. 3a and g, green region). Alanine substitutions in both acidic regions or the invariant region (183–188A mutant) did not interfere with E1A pre-mRNA splicing (Fig. 4a). Note that as a result of their construction by *in vivo* Cre-mediated site-specific recombination with the Ψ5 vector (30), all of these E1A mutants and control wild-type (wt) E1A were expressed from viral genomes that also contain an inactivating insertion of a *loxP* site in the C-terminal region of the E1B-55K coding region and a deletion of most of E3.

Viral promoter activation was assayed during infection of primary human bronchial/tracheal epithelial cells (HBTECs), derived from the natural Ad5 host tissue. We first analyzed the consequences of mutations in the DM, since deletion of both of the mutated regions in the DM was required to eliminate the interaction of E1A with p300 in the *in vivo* colocalization assay. HBTECs grown to confluence and arrested in G_1_ were infected with Ad5 mutants expressing either wt or mutant E1As. When infected at equal multiplicities of infection (MOIs) of 20, we observed that HBTECs expressed considerably more wt than DM E1A protein (data not shown), possibly due to differences in protein stability caused by the introduced mutations. Therefore, in order to achieve equal levels of wt and DM E1A protein, cells were infected with the wt virus at an MOI of 5 and diluted with the E1A deletion mutant *dl*312 at an MOI of 95. Alternatively, cells were infected with the DM virus alone at an MOI of 100. Under these conditions, the levels of wt and mutant E1A proteins were similar at 12 and 18 h postinfection (p.i.) (Fig. 4b). Further, dilution of the wt virus with dl312 gives the same concentration of adenovirus DNA template for all viral genes other than E1A compared to infections with the DM virus alone. Total RNA was isolated during the early phase of infection at 6, 12, and 18 h p.i. The late phase, determined by the onset of viral DNA replication, begins ∼24 h p.i. in these cells (data not shown). Viral RNAs transcribed from the early promoters E2early, E3, and E4, as well as late region L3 RNA, including hexon mRNA, were quantified by qRT-PCR (Fig. 4c). An unanticipated and remarkable result from these assays is that transcription from the different viral early promoters responded differently to the DM E1A multialanine substitutions.

Dramatic decreases in expression by the E1A DM were observed for RNAs transcribed from the E2 and E4 promoters, especially at 12 h p.i. (Fig. 4c). In contrast, no significant decrease in E3 RNA expression was observed at 12 h p.i., and only a modest (∼50%) decrease was observed at 18 h p.i. (Fig. 4c). The very low level of L3 RNA in the DM-expressing cells was similar in the coinfection of wt E1A and *dl*312 viruses at 12 h p.i. and was only very modestly decreased at 18 h p.i. While E2, E3, and L3 RNA increased between 12 h and 18 h p.i. in response to wt E1A, E4 RNA nearly reached a plateau at 12 h (Fig. 4c). This may be due to a decrease in the rate of E4 transcription between 12 and 18 h p.i., to changes in the stability of E4 RNA, or both.

Interpretation of expression levels for E1A and E1B are complicated by the 20-fold higher E1A template concentration in the DM-infected cells than the coinfection. *dl*312 is deleted for sequences from −50 relative to the major E1A transcription start site, including the TATA box, to −281 relative to the E1A poly(A) site, including all of the major E1A splice sites (1, 31). Any transcripts detected from the *dl*312 template with primers near the 3′ end of E1A are likely to have a different stability than full-length E1A mRNAs transcribed from the wt and DM templates. This would make a comparison of the steady-state level of the E1A RNAs difficult to interpret in terms of the activity of the E1A promoter. Similarly, deletion of E1B control elements far upstream of the E1A poly(A) site within the E1A 3′ exon (32), as well as stimulation of E1B transcription by read-through transcription from E1A (33), also complicate interpretation of results for E1B.

**FIG 5:**
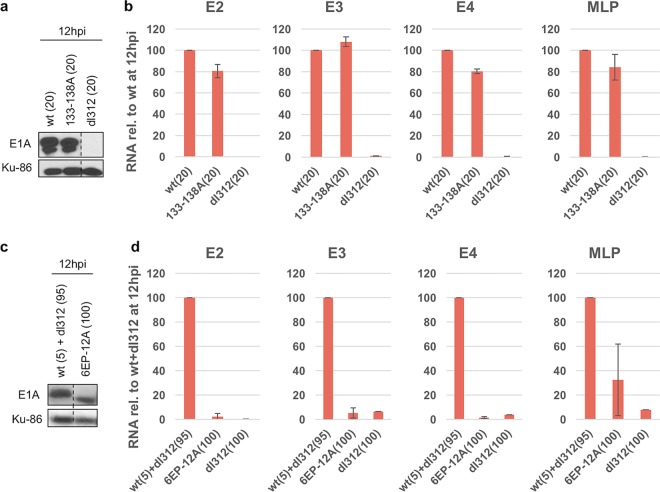
Activation of early viral promoters and MLP by wt and single acidic region mutant E1As. (a) Western blot showing wt and 133–138A mutant large E1A protein levels at 12 h p.i. HBTECs were infected with wt or 133–138A E1A-expressing virus at MOIs of 20. Ku-86 is a loading control. (b) Relative E2, E3, E4, and L3 RNA levels in infected HBTECs at 12 h p.i., as assayed by qRT-PCR. Cells were infected with the wt and 133–138A E1A-expressing virus at the same MOIs as those described for panel a. Values are plotted relative to those of the wt at 12 h p.i. Data are represented as averages ± SD from three independent experiments. (c and d) As described for panels a and b, except the 6EP-12A mutant was used. For experiments using this mutant, HBTECs were coinfected with the wt E1A-expressing virus at an MOI of 5 and *dl*312 at an MOI of 95 or infected with the 6EP-12A mutant E1A-expressing virus alone at an MOI of 100.

We also analyzed the effects of alanine substitutions in the individual acidic regions N terminal and C terminal to CR3 [aa 133 to 138, as well as the (EP)_6_ repeat, aa 189 to 200] (Fig. 5a to d). Deletions of each of these regions individually decreased luciferase activation ∼50% in the transient-transfection assay (Fig. 3b, compare 133-205, 133-189, 140-205, and 140-189). However, in infected HBTECs expressing equal wt and mutant E1A protein at 12 h p.i., multialanine substitutions in the 6EP region just C terminal to CR3 (6EP-12A) had a much greater effect on E2, E3, and E4 transcription than mutation of the acidic region aa 133 to 138 (DDEDEE) just N terminal to CR3(133–138A) (Fig. 5a to d).

### E1A amino acids 183 to 188 contribute to activation of early promoters E2, E3, E4, and MLP by binding multisubunit mediator complexes.

**FIG 6:**
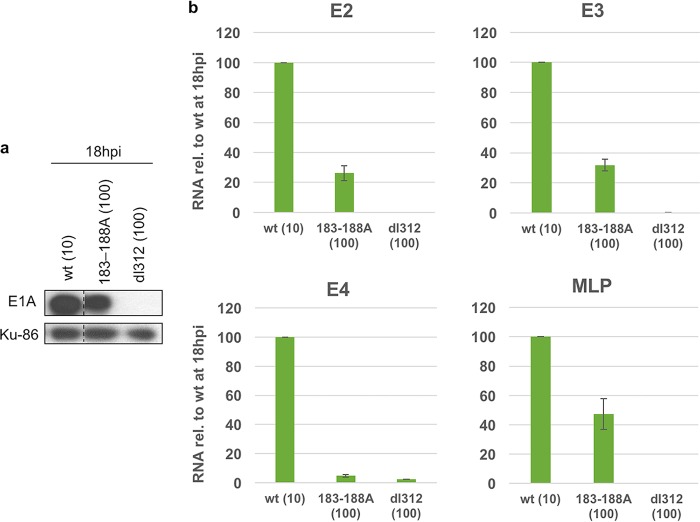
Activation of early viral promoters and MLP by wt and 183–188A mutant E1A. (a) Western blot showing wt and 183–188A large E1A protein levels at 18 h p.i. HBTECs were infected with the wt E1A-expressing virus at an MOI of 10 or with the 183–188A mutant-expressing virus at an MOI of 100. Ku-86 protein is a loading control. (b) Relative E2, E3, E4, and L3 RNA levels in infected HBTECs at 18 h p.i., as assayed by qRT-PCR. HBTECs were infected with the wt and 183–188A E1A-expressing virus at the same MOIs as those for panel a. Values are plotted relative to those of the wt at 18 h p.i. Data are represented as averages ± SD from three independent experiments.

An additional Ad5 E1A mutant was constructed with alanine substitutions in the aa 183 to 188 region (VYSPVS), corresponding to a sequence found just C terminal to the Zn-finger region in large E1A proteins that is precisely conserved in all primate adenoviruses (3). This invariable E1A sequence is in the region required for binding multisubunit mediator complexes, as assayed by colocalization with both YFP-MED23 and YFP-MED6 at the *lacO* array in CHO-A03.1 cells (Fig. 3c and d and Table 1). A serine residue in this region (aa 185) was left unsubstituted to avoid mutation of bases required for RNA splicing at the E1A 3′ splice site. In fact, the 183–188A E1A mutation had little effect on the ratio of unspliced E1A pre-mRNA to the spliced E1A mRNAs or the relative ratio of the 12S to 13S E1A mRNAs (Fig. 4a). Early viral gene expression in infected HBTECs expressing equal wt and 183-188A mutant E1A protein levels was assayed by qRT-PCR at 18 h p.i. (Fig. 6a). As expected, the E1A 183–188 region was required for most E1A activation of E2, E3, E4, and, to a lesser extent, the MLP (Fig. 6b). This is consistent with the requirement of this region for binding multisubunit mediator complexes (Fig. 3d and Table 1) and its crucial function in the transient-transfection assay (Fig. 3b, compare 133-189 to 133-178).

Taken together, these experiments indicate that the acidic region C terminal to CR3, the (EP)_6_ repeat, named AR1, for auxiliary region 1, by Ström et al. (17), contributes to E1A activation of early promoters E2, E3, and E4 during viral infection of human respiratory epithelial cells through an interaction with p300 and most likely the closely related host protein CBP. Further, E1A(aa 183–188) is essential for E1A activation of the early viral promoters E2, E3, and E4 and additionally the low MLP activity early in infection, probably because they are required for E1A to interact with multisubunit mediator complexes.

### Differences in E1A mutant-induced histone H3 acetylation at the early adenovirus promoters and their influence on RNA polymerase II preinitiation complex assembly and transcription.

**FIG 7:**
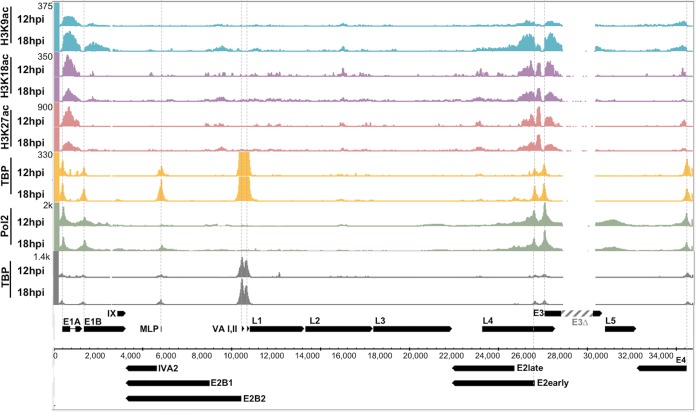
H3K9ac, H3K18ac, H3K27ac, TBP, and Pol II associated with the Ad5 genome. Genome browser plots of ChIP-seq normalized sequence tags in HBTECs infected with the wt E1A-expressing virus at an MOI of 20. Dotted lines indicate TSSs for early regions (E1A, E1B, E2early, E3, and E4), MLP, and VA I and II. ChIP-seq data for TBP are also shown at the bottom plotted on an expanded *y* axis (gray).

Because p300/CBP is primarily responsible for mediating the acetylation of histone H3 at lysines 18 (H3K18) and 27 (H3K27) (5, 8, 9), we performed ChIP-seq for these modified histones and aligned reads to the Ad genome in HBTECs infected with virus expressing wt E1A at an MOI of 20 at 12 and 18 h p.i. Clear H3K18ac and H3K27ac peaks at the E1A, E2early, and E3 promoters and smaller peaks at the E4 promoter were observed, primarily downstream of the transcription start sites (TSSs) (0 to +1 kb) (Fig. 7). These peaks coincided with GCN5/PCAF-mediated H3K9ac, which is also generally enriched around TSSs of active genes (34) (Fig. 7). Peaks for H3K9ac were also observed at the E1B promoter, and smaller peaks for H3K18/27ac were observed within the gene body of E1B. The time course of H3K9ac at some genes was slower than that of H3K18/27ac. At E1A, E1B, E2early, and E3, H3K9ac increased from 12 to 18 h p.i., whereas H3K18/27ac was maximal at 12 h p.i. The E2early and E3 TSSs demonstrated a clear absence of acetylated H3, indicating nucleosome-depleted regions upstream of the highly transcribed E2early and E3 TSSs (35), where preinitiation complexes have assembled, displacing nucleosomes. A single, high peak of H3K18/27ac was observed between the E2early and E3 TSSs (Fig. 7), probably representing a single acetylated nucleosome between them.

To assay Pol II preinitiation complex (PIC) assembly, we also did ChIP-seq for TBP and Pol II. The TBP subunit of TFIID is the first protein to interact with a TATA box promoter during PIC assembly, while Pol II is one of the last (36). We clearly detected peaks for TBP and Pol II at the active early promoters E1A, E1B, E2early, E3, and E4. At the MLP, TBP association was detectable at 12 h p.i., with a larger peak at 18 h p.i. A Pol II peak at the MLP became visible at 18 h p.i. The ChIP-seq signal for TBP at the two adenovirus genes transcribed by RNA polymerase III, VAI and VAII, was approximately five times higher than the signal for TBP at the early promoters transcribed by RNA polymerase II (Fig. 7, TBP track with expanded *y* axis at the bottom in gray).

**FIG 8:**
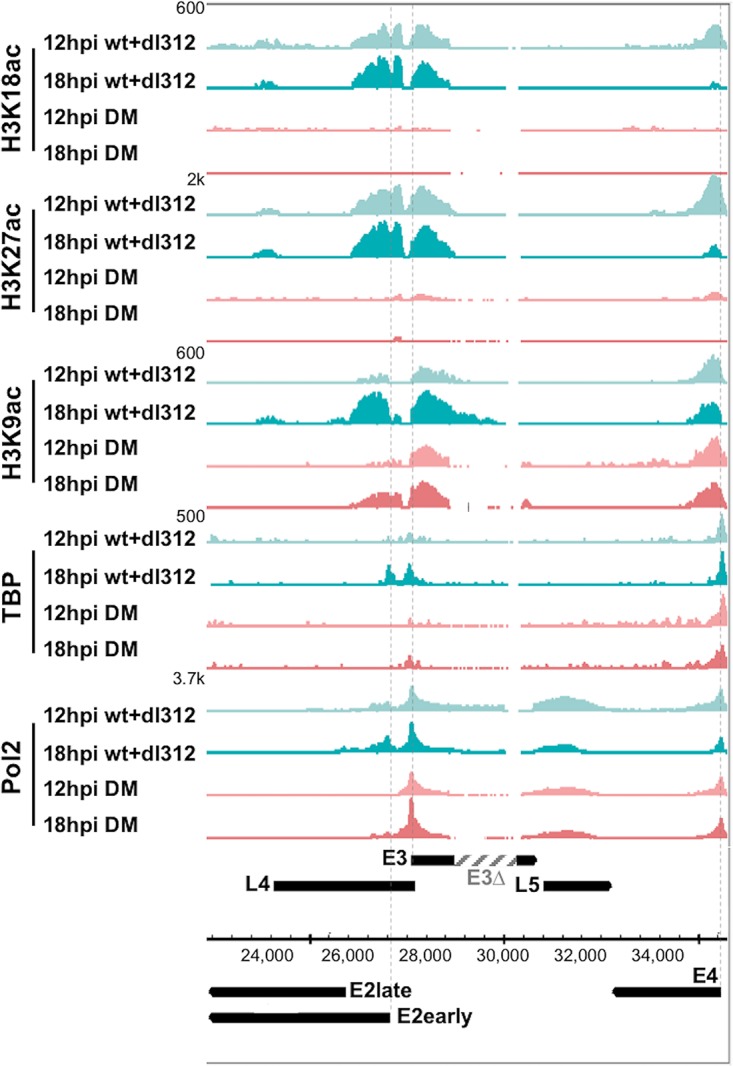
Defective H3 acetylation and PIC assembly by DM E1A at early viral promoters. Genome browser plots of H3K9ac, H3K8ac, H3K27ac, TBP, and Pol II ChIP-seq normalized sequence tags in HBTECs coinfected with the wt E1A-expressing virus at an MOI of 5 and *dl*312 at an MOI of 95 or infected with the DM vector alone at an MOI of 100. Dotted lines indicate TSSs for early regions E2early, E3, and E4.

To determine if the interaction of p300 with the E1A activation domain influences E2, E3, and E4 transcription via acetylation of histone H3 during infection, we also performed ChIP-seq for H3K18ac, H3K27ac, and H3K9ac in virus-infected HBTECs expressing equal concentrations of wt and DM E1A protein at 12 and 18 h p.i. We observed dramatic decreases in H3K18/27ac peaks at the E2early, E3, and E4 promoters in cells expressing DM E1A compared to wt E1A (Fig. 8). ChIP-seq for H3K9ac revealed no decrease in H3K9 peaks at the E3 and E4 promoters and a decrease at the E2early promoter to about 50% of the control level.

Pol II and TBP ChIP-seq experiments were subsequently performed to determine if these differences in H3 acetylation were associated with differences in PIC assembly on the early viral promoters. Striking decreases in TBP and Pol II association induced by DM E1A compared to wt E1A correlated well with the observed decreases in H3K18/27ac at the E2early promoter. Apparently, the failure of the E1A DM to induce H3K18/27 acetylation at the E2early promoter completely blocked RNA polymerase II PIC assembly on the E2early promoter. In the same cells infected with the E1A DM and as discussed above in relation to the E2early promoter, H3K18/27ac also was virtually eliminated at the E3 promoter, with a TSS only 418 bp to the right of the E2early TSS. However, in contrast to the nearby E2early promoter, at the E3 promoter TBP association was only slightly reduced compared to that of cells expressing the same concentration of wt E1A, and there was no apparent decrease in RNA polymerase II association. These ChIP-seq data correlated well with the much greater effect of the E1A DM mutations on expression of E2early RNA compared to that of E3 RNA (Fig. 4c).

Surprisingly, and of considerable interest, TBP and Pol II association with the E4 promoter were only minimally decreased by the DM mutations (Fig. 8), even though E4 RNA expression by the DM was nearly eliminated (Fig. 4c). These results suggest that the DM mutations block E4 transcription at a step subsequent to PIC assembly. Together these results demonstrate that the E2early promoter is regulated in part by p300/CBP-mediated acetylation of H3K18/27, which are required for a high level of Pol II PIC assembly, while H3K9ac appears to be sufficient for PIC assembly and moderate transcription at the E3 promoter. Interestingly, H3K18/27ac at the E4 promoter mediated by the p300 interaction with the E1A activation domain appeared to stimulate a step in transcription subsequent to TBP and Pol II association.

## DISCUSSION

Although there is a well-established correlation between promoter and enhancer region histone N-terminal tail lysine acetylation and transcriptional activity (34), the mechanism(s) by which acetylation of histone lysines stimulates transcription is not well understood in most cases (37). The results presented here on regulation of adenovirus transcription by large E1A demonstrate that promoter region H3K18/27ac is required for different steps in gene activation at different adenovirus promoters.

Using colocalization of fluorescently labeled proteins in nuclei *in vivo*, we confirm an interaction between the strong E1A activation domain included in E1A(aa 133–205) and the acetyltransferase p300, as reported by Pelka et al. (25), on the basis of coimmunoprecipitation from transiently transfected cell extracts. We further mapped the partially redundant regions of E1A that bind p300 *in vivo* and show that this interaction with p300 contributes to H3K18/27ac, Pol II PIC assembly, and transcriptional activity of early viral promoters during viral infection of primary airway epithelial cells but to significantly different extents at each early promoter.

The regions of E1A required for the association of E1A(aa 121–223) with p300 were mapped to two acidic peptides flanking CR3, aa 133 to 138 (DDEDEE) and aa 189 to 200 (EPEPEPEPEPEP). This EP repeat was previously shown to contribute to maximal E1A activation of the early adenovirus promoters in transient-transfection assays with native E1A as opposed to Gal4DBD-E1A fusions and was named auxiliary region 1 (16, 17). The negative charge of the (EP)_6_ region is primarily responsible for its activity in this assay (17). Reducing the number of EP repeats reduced activation, a (DP)_6_ repeat and alanine substitutions of the prolines had activity similar to that of (EP)_6_, and an (AP)_6_ substitution was inactive (17). Deletion of both acidic regions resulted in the loss of p300 binding in our *in vivo* colocalization assay and ∼10-fold decreased activity in our transient-transfection reporter assays using *lacI* fusions and a *lacO* reporter (Fig. 3a, compare 140-189 to 121-223). Individually, either of the two acidic regions was sufficient for p300 association in our *in vivo* colocalization assay. However, the transient-transfection assay revealed that deletion of either region caused an ∼2-fold decrease in activation function (Fig. 3b and e). In contrast, infection of cells with viruses containing multialanine substitutions in either or both of the two acidic regions (133–138A and 6EP-12A mutants) showed that during infection, mutation of the 189–200 EP repeat, equivalent to auxiliary region 1 (16, 17), was primarily responsible for the decrease in transcriptional activation of E2, E3, and E4 by DM E1A (Fig. 4). The greater effect of the EP repeat on activation may also extend to E1A proteins from human adenoviruses other than the respiratory adenoviruses in species C, since they contain a considerably longer peptide linker between the N-terminal acidic region and the beginning of CR3 (3). It is also interesting that these two acidic regions are also present in small e1a, although they are likely positioned differently relative to each other in the absence of the intervening CR3. Despite the presence of these two acidic regions in small e1a (Fig. 1), in coimmunoprecipitation assays, small e1a binds p300 only through the well-characterized interaction between the e1a N terminus and CR1 and the p300 equivalent of the CBP TAZ2 domain (5, 7, 25).

### A revised model for functions of E1A activation subdomains.

Earlier, we found that the C_4_-Zn-finger region of large E1A(aa 140–178) (28) is required for E1A binding to multisubunit mediator complexes required for *in vitro* transcriptional activation by Gal4 fusions to E1A and other activation domains (20). The same E1A Zn-finger region amino acids required for binding to multisubunit mediator complexes also were required to bind free MED23 mediator subunits in excess over multisubunit mediator complexes in HeLa nuclear extracts (20). These results, plus the observation that overexpression of MED23 inhibits, or squelches, E1A activation and not activation by Gal4-VP16 led to the conclusion that E1A binds multisubunit mediator complexes by binding the MED23 mediator subunit. This conclusion was further supported by knockout of the mouse *Med23* gene, which generated cells that supported activation by the VP16 activation domain but not the E1A activation domain (38). Further, *Med23^−/−^* murine embryonic fibroblasts (MEFs) infected at low MOI with wt mouse adenovirus I (MAVI) failed to express the MAV early mRNAs, similar to the phenotype observed for E1A-minus MAVI in wt MEFs (39).

Here, we report that the C-terminal six amino acids of CR3 (aa 183 to 188) that are invariable in all primate adenoviruses (3) are necessary, in addition to the MED23-binding Zn-finger, for E1A binding to multisubunit mediator complexes, as opposed to monomeric MED23. To assay association of E1A deletions with multisubunit mediator complexes *in vivo*, we analyzed nuclear colocalization of LacI-CFP-E1A fragment fusions with YFP-MED6 at the *lacO* array in CHO-A03.1 cells (Fig. 2g to l). MED6 is located in the mediator head domain, distant from MED23 in the tail domain (22). Consequently, association of an E1A deletion with both YFP-MED6 and YFP-MED23 (Fig. 3c and d and Table 1) implies that the retained region of E1A binds multisubunit mediator complexes, as shown for E1A(aa 121–223) by direct biochemical analysis (20). The requirement for aa 183 to 188 for the E1A interaction with multisubunit mediator complexes explains the requirement of this region of CR3 for transcriptional activation without invoking a promoter targeting function for this region (14). During viral infection, E1A(aa 183–188) is required for activation of early promoters E2, E3, and E4 (Fig. 6). Our results described above suggest that this is because this invariant region of E1A is required for E1A interaction with multisubunit mediator complexes.

Amino acid substitutions in the completely conserved C-terminal region of CR3 are defective for activation of the viral E3 promoter in a transient-transfection assay (18). They are also dominant negative over wt E1A for activation. In contrast, while mutants in the Zn-finger region are also defective for E3 activation, they are not transdominant (18). These observations supported the model that the Zn-finger region of CR3 binds a limiting factor required for activation of the early viral promoters, while the C-terminal ∼10 amino acids of CR3 target the resulting complex to the early viral promoters (14, 18). However, our results provide an alternative explanation for the dominant-negative phenotype of the C-terminal CR3 mutants. We observed that the E1A Zn-finger region alone binds MED23 (Fig. 3c and Table 1, fragment 140-178), and that the invariant region of E1A immediately C terminal to the Zn-finger is additionally required to bind multisubunit mediator complexes (Fig. 3c and d and Table 1). Based on these results, we propose that mutants in the invariant region compete with wt E1A for binding MED23 in multisubunit mediator complexes, because they have an intact Zn-finger region that binds MED23 (Fig. 3a, blue, 140-178). However, they are defective for interactions with other mediator subunits required for activation and are therefore dominant negative over wt E1A. In contrast, mutations in the Zn-finger subdomain are recessive (18) because they do not compete with wt E1A for binding multisubunit mediator complexes via their MED23 subunit.

### The large E1A activation domain.

The original model that divided the CR3 region into an activation domain and a promoter-binding domain came from studies of Gal4 fusions to E1A fragments in transient-transfection assays (14, 15). Those studies reported, for the first time, that E1A(aa 121–223) has high activation domain activity when fused to the Gal4 DNA-binding domain. N-terminal deletions of E1A(aa 121–223) fused to the Gal4 DNA-binding domain from E1A(aa 121–140) retained activation domain activity in the transient-transfection assays, as did C-terminal deletions to aa 178. Consequently, the activation domain was presumed to map to the Zn-finger region between aa 140 and 178 (Fig. 3a, blue). However, we found that a LacI fusion to E1A(aa 140–178) did not activate a *lacI* reporter in a transient-transfection assay (Fig. 3b). Similar results were observed for a Gal4-DBD fusion to E1A(aa 140–178) versus a fusion to E1A(aa 121–223) and a reporter with five Gal4 binding sites upstream of the E1B promoter (A. J. Berk, unpublished results). This apparent discrepancy is explained by the two acidic regions flanking CR3, one from aa 133 to 138 and another from aa 189 to 200, that are functionally redundant in the transient-transfection assay (Fig. 3b). As a consequence, the E1A N-terminal deletion to 140 retains the C-terminal acidic region from aa 189 to 200, while the C-terminal deletion to 189 retains the functionally compensating N-terminal acidic region from aa 133 to 138.

The earlier model of an N-terminal activation and a short C-terminal promoter binding domain in CR3 was further supported by experiments involving fusions of known functional domains of the yeast Gal4 activator and the HSVI VP16 immediate-early activator to inactive deletions of E1A (14, 15). These experiments showed that E1A(aa 121–223) activated the Ad5 E4 promoter, that mutations in the Zn-finger region of this part of E1A prevent activation of the E4 promoter, and that these Zn-finger region mutations can be compensated for by fusion of the VP16 activation domain. These results suggested that the Zn-finger region alone functions as an activation domain whose function could be replaced by the VP16 activation domain (14, 15). However, our observation that LacI-E1A(140–178) does not activate transcription indicates that the E1A Zn-finger region alone does not function as an activation domain. Rather, E1A(aa 179–189) is additionally required for activation domain activity because it is required for E1A binding to multisubunit mediator complexes. The defect in binding to multisubunit mediator complexes of E1A mutants in the aa 140 to 178 region can be compensated for by fusion of the VP16-AD, since it also interacts with multisubunit mediator complexes (40, 41).

As a Gal4 fusion, E1A(aa 121–223) stimulates the assembly of a PIC on a template with Gal4-binding sites (24). Presently, however, the mechanism by which the mediator interaction with native E1A (i.e., not fused to a DNA-binding domain) stimulates transcription remains unclear. We propose that wt E1A and an associated multisubunit mediator complex are targeted to early adenovirus promoters through interactions between activation domains of the cellular activators bound to early viral promoters (42) and multisubunit mediator complexes. The interactions of TFIID and the Pol II general transcription factors with both viral promoters and the mediator probably also contribute to association of mediator complexes with associated E1A to early viral promoters. The low level of transcription from the early viral promoters in the absence of E1A is greatly stimulated by the high-affinity interaction of the E1A Zn-finger region with MED23 as well as additional interactions of E1A(aa 183–188) that result in binding to multisubunit mediator complexes. One possibility is that these E1A-mediator interactions induce a conformational change in the mediator that favors its association with Pol II, as has been proposed for other activation domains (22, 43, 44).

### The E1A interactions with p300 do not greatly stimulate early transcription from the MLP.

At 18 h p.i., L3 is expressed at very low levels compared to the late phase of infection, when virus-encoded transcriptional activators bind in the first intron to stimulate transcription from the MLP several hundredfold (45). However, we observed ∼20-fold activation by wt E1A of the low level of L3 RNA expression at 18 h p.i., presumably from the MLP. This can be seen by comparing L3 RNA in cells infected with a mixture of the wt E1A-expressing virus and *dl*312 virus and cells infected with *dl*312 alone (Fig. 4c, MLP). However, this activation was not greatly dependent on the interaction of the E1A AD with p300, because it was not decreased by the E1A DM mutations at 12 h p.i. and was reduced only ∼50% at 18 h p.i. (Fig. 4c). Consistent with this, H3 acetylation was not observed at the MLP at 12 and 18 h p.i. with Ad expressing wt E1A (Fig. 7).

### TBP association at Pol II and Pol III promoters on the adenoviral genome.

It is remarkable that the ChIP-seq signal for TBP at the two adenovirus genes transcribed by RNA polymerase III, VAI and VAII, was approximately five times higher than the signal for TBP at the early promoters transcribed by RNA polymerase II (Fig. 7, bottom, gray). This indicates that at the time of cross-linking, more viral DNA molecules are bound by the RNA polymerase III TBP-containing initiation factor TFIIIB than are bound by TFIID at the promoters of viral genes transcribed by Pol II. However, another likely possibility is that TBP is much more readily indirectly cross-linked to promoter DNA as a subunit of TFIIIB than as a subunit of TFIID. The complexes of TFIIIB with tRNA and 5S rRNA promoter DNA are stable to high salt and high concentrations of heparin (46). In contrast, RNA polymerase II preinitiation complexes are disrupted by 0.5 M KCl (47). This implies that much more of the TFIIIB protein, comprised of TBP, BRF, and BDP subunits, contacts DNA in the Pol III preinitiation complex than does TFIID and other Pol II general transcription factors in a Pol II PIC. Further, in the crystal structure of a TBP-BRF-DNA complex (48), BRF interacts extensively with the convex top of TBP, and BRF helix 25 (absent in TFIID) extends along the downstream promoter DNA for six base pairs. This may increase the opportunity for cross-linking TBP to DNA indirectly through cross-links to BRF that is cross-linked to DNA.

### Histone H3 acetylation at viral early promoters.

Histone lysines H3K18 and H3K27 are acetylated primarily by p300 and its closely related paralog, CBP (8, 9). We analyzed acetylation at these sites on histones associated with the viral genome early during infection by performing ChIP-seq in HBTECs infected with Ad5 mutants expressing wt E1A proteins and mapping the immunoprecipitated DNA sequences to the viral genome (Fig. 7). We observed significant peaks of H3K18/27ac at the E1A, E2early, E3, and E4 promoters at 12 and/or 18 h p.i. but not at the E1B or MLP promoters (Fig. 7). H3K18/27ac at the E2early, E3, and E4 promoters was greatly decreased following infection with the E1A DM defective for the E1A activation domain-p300 interaction. These results indicate that H3K18/27 acetylation at these promoters requires targeting p300/CBP to the promoters through their interaction with the acidic regions flanking CR3 [E1A(aa 133–138) and (EP)_6_ repeat, aa 189 to 200]. The virtual absence of H3K18/27ac at the E2early promoter following infection with the DM correlated with an absence of TBP and Pol II association, suggesting that H3K18/27ac by p300/CBP is required for E1A stimulation of Pol II preinitiation complex assembly *in vivo* at this promoter (Fig. 8). Residual H3K9ac at the E2early promoter in cells expressing DM E1A was not sufficient for high levels of Pol II PIC assembly. These observations are consistent with the model that hypoacetylated nucleosomes inhibit the association of TFIID and other Pol II general transcription factors with promoter DNA, and that histone acetylation makes the promoter elements more accessible to the GTFs. Histone tail acetylation at these early viral promoters also may generate binding sites for proteins that stimulate transcription, including proteins with bromodomains that bind acetylated lysines (49).

In contrast to the E2early promoter, H3K18/27ac is not required for significant levels of PIC assembly or transcription from the nearby E3 promoter. (The E2early and E3 TSSs are separated by only 418 bp.) H3K9ac in the absence of H3K18/27ac may be sufficient for PIC assembly at the E3 promoter.

While hypoacetylated H3K18/27ac was observed at the E4 promoter in cells expressing DM E1A, high levels of H3K9ac as well as TBP and Pol II association were observed. This suggests that H3K18/27ac is not required for PIC assembly at the E4 promoter. However, H3K9ac at the E4 promoter, which continues to occur in cells expressing the E1A DM, may be sufficient for Pol II PIC assembly. Surprisingly, in cells infected with the E1A DM, we noted a clear discrepancy between the TBP and Pol II ChIP-seq signals at the E4 promoter and the qRT-PCR data for E4 mRNA. Pol II and TBP peaks were not reduced at the E4 promoter in DM-infected cells (Fig. 8), yet H3K18/27ac and E4 mRNA expression by the DM were greatly decreased (Fig. 4c). These results lead us to speculate that H3K18/27ac mediated by the E1A-p300/CBP interaction at the E4 promoter regulates elongation of the polymerase, rather than Pol II PIC assembly, as occurs at the E2early promoter. This model of E1A stimulation of Pol II elongation from the E4 promoter may explain why H3 acetylation occurs primarily downstream from the TSS (Fig. 7). E1A has been previously shown to promote transcriptional elongation at early viral genes by recruiting the hPaf1 complex (50). Perhaps promoter region H3K18/27ac is required for E1A to stimulate hPaf1-dependent elongation at the E4 promoter.

H3K9ac peaks were present at the E2early, E3, and E4 promoters in addition to H3K18/27ac and were decreased after infection with the DM at the E2early promoter (Fig. 8). This indicates that p300 recruitment by E1A and/or H3K18/27 acetylation regulate H3K9 acetylation at the E2early promoter. This decrease in H3K9ac may be because H3K9 is less accessible to acetyl transferases in the absence of H3K18/27ac or because PCAF/hGCN5, the acetyl transferases primarily responsible for H3K9 acetylation (9), are normally recruited to the E2early promoter through interactions with H3K18/27ac.

These patterns of H3 acetylation at early viral promoters in response to multialanine substitutions of subdomains of the E1A activation domain indicate that H3K18/27ac by p300/CBP is required for maximal H3K9 acetylation and the assembly of Pol II PICs at the E2early promoter. In contrast, Pol II PIC assembly on the E3 and E4 promoters is associated with H3K9ac but not H3K18/27ac. In the case of E3, this is sufficient for PIC assembly and moderate transcription. At the E4 promoter, the E1A activation domain-p300/CBP interaction is not required for TBP and Pol II association but is required for H3K18/27ac and transcription. This may be because elongation of paused Pol II at the E4 promoter is dependent on H3K18/27ac. By eliminating the association of the E1A activation domain with p300, we have revealed striking diversity in transcriptional regulation of the viral genome early in infection, including nuanced roles for p300/CBP at each early viral promoter.

## MATERIALS AND METHODS

### Plasmids.

The plasmids used for *in vivo* colocalization assays and luciferase reporter transient-transfection assays were constructed from plasmid NYE127 (29) containing the F9-polyomavirus early promoter driving expression of the SV40 T-antigen nuclear localization sequence (NLS)-EYFP-lac repressor fusion fused to fragments of large E1A [e.g., NLS-EYFP-LacI-E1A(aa 121–223)] (27). The five C-terminal amino acids of LacI are deleted in these expression vectors so that the fusion proteins form LacI dimers, not tetramers (26). PCR amplification of regions encoding the indicated large E1A amino acids were cloned into the NYE127 derivatives via a SacI restriction site and verified by sequencing. For cyan fluorescent protein (CFP) or mCherry-labeled proteins, enhanced yellow fluorescent protein (EYFP) was replaced by ECFP or mCherry generated by PCR and confirmed by sequencing.

### Confocal microscopy of transfected CHO-A03.1 cells.

CHO-A03.1 cells (26) were plated on fibronectin-coated 6-well plates and transfected using Lipofectamine 2000 (Invitrogen) with 2 μg of expression vector for LacI-CFP-E1A or LacI-mCherry-E1A and 2 μg of expression vector for YFP-MED6, YFP-MED23, or YFP-p300 (5, 27). After 48 h, cells were fixed in 2% paraformaldehyde, washed in 1× phosphate-buffered saline (PBS), and stained with 4′,6-diamidino-2-phenylindole (DAPI) in 0.05% Triton X-100 in PBS for 20 min. Cells were mounted onto slides and imaged for colocalization of CFP or mCherry and YFP using a Leica TCS SP2 AOBS single-photon confocal microscope using a 63×, 1.4-numerical-aperture oil immersion objective.

### Transient-transfection reporter assays.

Transfection was done into CHO-K1 cells, the parental cell for A03.1 cells used in the fluorescence microscopy colocalization assay (26). Seventy percent confluent 12-well culture plates were transfected with 100 ng expression vectors for NLS-mCherry-LacI fused to the indicated fragments of large E1A (Fig. 3a), 300 ng NYE107b luciferase reporter plasmid with eight *lacO* sites upstream of the Ad2 E1B promoter driving firefly luciferase expression (29), and 300 ng pRL-TK (Promega) per well. Cells were cultured for 24 h, extracts were prepared and assayed for firefly and Renilla luciferase activity using the Promega Dual-Luciferase assay kit, and firefly luciferase activity was normalized to Renilla luciferase activity.

### Construction of Ad5 mutants.

The wt Ad2 sequence from aa 1 to 3153 was cloned between the SfII site next to Ad5 nucleotide 1 and the StuI site at Ad5 nucleotide 3153 in plasmid pAdlox (30). Oligonucleotide-directed mutagenesis was used to substitute alanine codons for wt Ad2 E1A codons, as shown in Fig. 3g. The resulting plasmids were cut with SfiI and cotransfected into 293-CRE cells with Ψ5 DNA (30). Cre-mediated recombination between the *loxP* site in Ψ5 at base pair 3201 and the *loxP* site in the plasmids derived from pAdlox with the engineered E1A mutations generates genomes with inverted terminal repeats and the left end packaging sequences capable of replicating and being packaged into virions in 293 cells (30). In addition to the engineered E1A mutations, all of these mutants have a substitution in E3 (30) and an out-of-frame insertion of a *loxP* site near the C-terminal end of the E1B-55K coding region that probably inactivates E1B-55K function (51). The virus expressing wt E1A in these studies was constructed in the same way using a pAdlox plasmid with wt Ad2 E1A sequence.

### Cell culture.

Human bronchial/tracheal epithelial cells (HBTEC; catalog number FC-0035, lot number 02196; Lifeline Cell Technology) were grown at 37°C in a BronchiaLife medium complete kit (LL-0023; Lifeline Cell Technology) in a 5% CO_2_ incubator until they reached confluence. Cells were then incubated 3 days more without addition of fresh medium and were infected with the indicated Ad mutants under the conditioned medium.

### qRT-PCR.

Total RNA extracted from HTBECs using a PureLink RNA minikit (Ambion) was reverse transcribed with random hexamer priming using Superscript III (Invitrogen). RNA was treated with DNase I with a DNA-free kit (Ambion). Quantitative reverse transcription-PCRs (qRT-PCRs) were carried out with the Applied Biosystems 7500 real-time PCR system with FastStart universal SYBR green master mix (Roche). All values were normalized to 18S RNA levels.

### ChIP-seq.

Preparation of cross-linked HBTEC chromatin, sonication, and immunoprecipitation was as described in reference 5. Sequencing libraries were constructed from 1 ng of immunoprecipitated and input DNA using the KAPA Hyper Prep kit (KAPA Biosystems) and NEXTflex ChIP-seq barcodes (Bio Scientific). Sequence tags were aligned to the Ad5 genome and normalized to the following formula: (number of Ad5-aligned reads in the input sample/number of human-aligned reads in the input sample) × (number of Ad5-aligned reads in the ChIP sample).

### Antibodies.

Antibodies included H3K9ac (07-352; Millipore) and H3K18ac (814), prepared and validated as described previously (52), as well as H3K27ac (39133; Active Motif), Pol2 8WG16 (sc-56767; Santa Cruz), TBP (C15200002; Diagenode), anti-e1a MAb M73 (53), and Ku-86 H-300 (sc-9034; Santa Cruz).

### Accession number(s).

DNA sequencing data related to Fig. 7 and 8 have been deposited in the Gene Expression Omnibus (GEO) under accession number GSE116772.
